# Short High-Intensity Interval Exercise for Workplace-Based Physical Activity Interventions: A Systematic Review on Feasibility and Effectiveness

**DOI:** 10.1007/s40279-023-01821-4

**Published:** 2023-02-25

**Authors:** Stefano Amatori, Carlo Ferri Marini, Erica Gobbi, Davide Sisti, Germana Giombini, Rosalba Rombaldoni, Marco B. L. Rocchi, Francesco Lucertini, Ario Federici, Fabrizio Perroni, Giorgio Calcagnini

**Affiliations:** 1 Department of Biomolecular Sciences, University of Urbino Carlo Bo, Via Dell’Annunziata 4, 61029 Urbino, Italy; 2 Department of Economics, Social Science, and Politics, University of Urbino Carlo Bo, Urbino, Italy

## Abstract

**Background:**

Workplace exercise interventions showed good results, but lack of time was often reported as a barrier to participation. To overcome this problem, several studies attempted to implement short high-intensity interval training (HIT) within the workplace.

**Objectives:**

The aim of this systematic review is to explore the feasibility and effectiveness of HIT interventions within the workplace setting.

**Data sources:**

A systematic literature search was conducted in PubMed and SPORTDiscus to identify articles related to HIT within the workplace.

**Study eligibility criteria:**

Only interventions that consisted of HIT programmes within the workplace and tested at least one physiological, psychological, or work-related outcome were included.

**Results:**

Seven studies (317 participants) met the inclusion criteria. HIT interventions lasted 6–12 weeks, with a frequency of 2–4 sessions/week and a duration of 8–30 min per session. Feasibility was qualitatively investigated in four studies, with key positive aspects reported for HIT time-appeal, the sense of competence driven by individual intensity, and improved intention to exercise; five studies reported adherence rates > 80%. Small-to-large effect sizes were reported for improvements in cardiorespiratory and muscular fitness. Small-to-medium effect sizes were reported for blood parameters and health-related quality of life.

**Conclusions:**

HIT interventions in the workplace showed limited effectiveness in improving health-related outcomes, while promising results regarding feasibility were reported, mainly due to the time-efficiency and the positive post-exercise psychosocial responses. However, further high-quality studies involving more participants are still needed to make firm conclusions on HIT effectiveness and feasibility compared to other types of exercise in this context.

**Supplementary Information:**

The online version contains supplementary material available at 10.1007/s40279-023-01821-4.

## Key Points

**Table:** 

Poor cardiorespiratory fitness and insufficient physical activity are well-known risk factors for negative physical and psychological health outcomes, compromising adult work performance.
High-intensity interval training (HIT) seems to represent a feasible strategy to overcome the barriers to physical activity participation within the workplace. Short (10–20 min) HIT interventions showed limited effectiveness in improving physiological and psychological outcomes.
Rigorous high-quality studies are still necessary to support the effectiveness of HIT interventions in the workplace setting and to quantify the economic impact of such health-promoting strategies.

## Introduction

Poor cardiorespiratory fitness (CRF) is associated with an increased risk of all-cause and cardiovascular mortality [1] and, along with insufficient physical activity, is a well-known risk factor for adverse physical and psychological health outcomes [2], affecting, among other things, adult work performance [3]. In particular, cardiovascular diseases, musculoskeletal disorders, hypertension, and depression-related illnesses are among the costliest conditions affecting employees, leading to increased presenteeism and absenteeism and thus loss of work productivity [3–5]. According to the World Health Organization [6], in 2016, 11.9 days of work per employee were lost on average due to sickness absenteeism. The reduction in absenteeism and the need for medical assistance are estimated to save a cost of US$2–3 per dollar invested in implementing effective health programmes for the workers [7].

Given the huge amount of time people spend at work, the workplace represents an ideal setting for health-promoting interventions based on lifestyle modifications. Employees' physical fitness and wellbeing play an important role in job satisfaction and productivity [8, 9], and in the past decades, several workplace health programmes (including physical activity, stress management, and healthy nutrition) have been implemented and evaluated, appearing efficacious in reducing cardiovascular risk, diminishing absenteeism, and thus improving work performance [10, 11]. Although the literature is controversial, owing primarily to methodological limitations (i.e. randomisation, poor compliance) [9], regular well-structured health-enhancing exercise routines within the workplace have been proposed as a potential solution to counteract the adverse effects of prolonged sitting time, sedentary behaviour, and monotonous and/or strenuous physical tasks [12]. A meta-analysis published by Prieske et al. [9] summarised the results of 17 randomised controlled trials (RCTs) of physical training in the workplace. Interventions included resistance training, endurance training, team-sports activities, or combined training, with most of the training sessions lasting between 30 and 60 min, at intensities ranging from low to vigorous. The main findings were that physical exercise in the workplace led to significant improvements in workers’ CRF and muscle endurance and power, particularly in white-collar workers. A relationship between training intensity and CRF improvements was also suggested, with higher gains following high-intensity training compared to moderate intensity [13]. These fitness gains could also be translated into fewer neck, shoulder, and back pain issues, which considerably impact work productivity, sickness absences, and work disability-related costs [14]. Indeed, a higher CRF was associated with a decreased risk of having a sickness episode [15].

Although the results of some physical activity interventions in the workplace seem promising, many initiatives still fail. Exercise programmes are generally not integrated into the work environment, are constrained to a “would be nice to have” add-on, and are often sacrificed when companies encounter financial problems [16]. Usually, wellbeing programmes in the workplace just evaluate the health gains of the employees without monitoring the economic return for the company, when employers need to recognise a financial benefit to support physical activity initiatives [16, 17]. Moreover, lack of time, work schedule conflicts, low perceived self-efficacy, and lack of motivation were reported to be the most important barriers to workplace exercise participation [18, 19]. Strategies to overcome these barriers include offering more opportunities to exercise throughout the workday and organising frequent group exercise classes [20]. Further, the variety of the exercise selection appears to be a key factor in facilitating exercise engagement, as a single exercise mode might not facilitate adherence or compliance [21]. In this context, time-efficient and enjoyable exercise modalities, such as high-intensity interval training (HIT) [22–24], could represent a strategy to overcome the perceived barriers to physical activity participation within the workplace [9]. HIT was defined as "either repeated short (< 45-s) to long (2–4 min) bouts of rather high-intensity exercise, or short (< 10-s) to long (20–30-s) all-out sprints, interspersed with recovery periods” [25]. Such a training modality was shown to be equally—if not more—effective for enhancing CRF and several other health-related markers as traditional moderate-intensity continuous exercise [26], with the advantage of being time-efficient and not necessarily requiring any equipment or a large space [27]. HIT can be incorporated into a daily routine and adopted in a home, school, or workplace setting [26], although its feasibility has been a subject of debate because of possible adverse affective responses [28]. Indeed, HIT has been criticised for its strenuous nature, which might undermine confidence in sustaining exercise behaviour over time, particularly in clinical or sedentary populations [28]. On the contrary, some evidence reported comparable, or even superior, enjoyment following HIT than after moderate-intensity exercise, possibly as a result of a positive interplay between effort and discomfort counteracted by time efficiency and continuously changing stimuli [22–24]. Therefore, since HIT can be performed in small groups and can be adapted to the fitness level, skills, and needs of everyone, it has been viewed as a viable alternative for workplace physical activity programmes.

In the last few years, a large number of studies have been published about the effectiveness of HIT on different populations; however, the evidence is usually derived from laboratory settings. To be transferred and adopted in the real-world, HIT interventions must show their feasibility and effectiveness in daily practice, with the constraints of limited time and resources [29]. Indeed, feasibility influences the effectiveness of such interventions in achieving the desired outcomes. Several studies have sought to apply HIT programmes among adult workers directly in the workplace to boost accessibility and time-appeal while also enhancing the employees' psychological and physical wellbeing. This systematic review aims to summarise the evidence about the feasibility and effectiveness of HIT interventions in the workplace setting for improving health- and work-related outcomes in adult workers.

## Methods

This systematic review was conducted following the Preferred Reporting Items for Systematic reviews and Meta-Analyses (PRISMA) 2020 statement [30].

### Literature Search

**Fig. 1:**
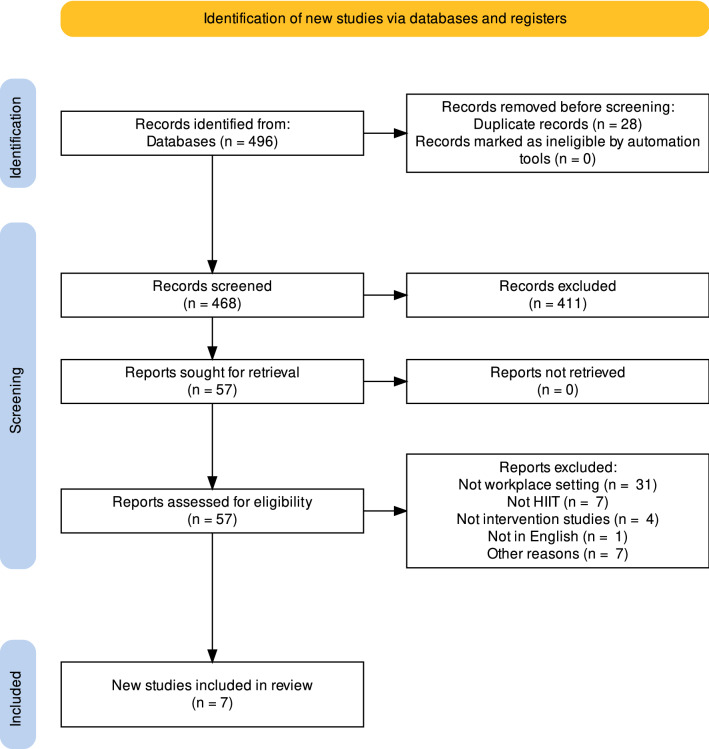
PRISMA flow diagram. Created with the ShinyApp by Haddaway et al. [31]. *HIIT* high-intensity interval training, *PRISMA* Preferred Reporting Items for Systematic reviews and Meta-Analyses

The systematic search and screening strategy were conducted in the online databases PubMed (Medline) and SPORTDiscus, in September–October 2021. The primary search syntax included elements about populations and interventions, as follows: ((“workplace”) OR (“corporate”) OR (“workplace wellness”) OR (“corporate wellness”) OR (“workplace setting”)) AND ((“high-intensity exercise”) OR (“high-intensity training”) OR (“high-intensity interval training”) OR (“HIIT”) OR (“HIT”) OR (“SIT”) OR (“HIIE”)). SIT and HIIE stand for “sprint interval training” and “high-intensity interval exercise”, respectively. A secondary search was conducted by cross-checking the reference lists of the selected studies. The study selection process is reported using the PRISMA flow diagram (Fig. 1) [31].

### Eligibility Criteria for Selecting Studies

Studies included in the review were limited to peer-reviewed papers written in English. The search strategy was not limited solely to RCT studies; in an attempt to better investigate feasibility, it was considered that the aim would be better pursued by including other study designs (pilot studies, pre-post, feasibility studies) to obtain a more comprehensive view of the topic. All titles and abstracts were initially screened according to the following inclusion criteria: (1) intervention studies; (2) conducted in the workplace setting; (3) including HIT protocols (defined as brief [< 10-s to 4-min] bursts of high-intensity exercise [> 85% of the maximal heart rate {HR_max_} or performed “all-out”], interspersed by recovery periods [25, 32], performed in activities involving large muscle mass [such as running, cycling, stair-climbing] [33]); and (4) considering either physical (body composition, CRF, muscle strength, blood pressure, haematochemical parameters), psychological (mental wellbeing, health-related quality of life [HR-QoL], stress, anxiety, motivation and self-efficacy to exercise), or work-related outcomes (job satisfaction, productivity). Studies were excluded if (1) the intervention did not involve workers/employees and (2) the exercise programme was not characterised as HIT.

### Data Extraction

The literature search and the inclusion/exclusion of relevant studies based on the titles were performed by the first author. Considering the eligibility criteria detailed above, the full texts of the remaining studies were reviewed, and two authors (SA and CFM) extracted data. Disagreements between the two authors were resolved through personal communications or by asking for the opinion of a third author (EG). Information was extracted from each paper following the PICO (i.e. participants, interventions, comparators, outcomes) framework: (1) participants: sample size, age, sex, employment, physical activity level (e.g. sedentary, active); (2) intervention: training type (e.g. cycling, stair-climbing) and modalities (e.g. sets, repetitions), training frequency (sessions/week), session duration and exercise intensity, intervention duration (weeks); (3) comparator: the presence of a control group performing other forms of exercise; (4) outcomes: physical outcomes (e.g. cardiovascular fitness, strength), psychological outcomes (e.g. quality of life, stress), work-related performance (e.g. work productivity). For each outcome, pre- and post-intervention data (mean and standard deviation) were extracted, and within-group Cohen’s effect sizes were calculated to compare the results of included studies (Online Resource 1, see the electronic supplementary material). In cases where a study reported confidence intervals (CIs), the standard deviation (SD) was calculated as:

$${\text{SD }} = \sqrt n \frac{{{\text{CI}}_{{{\text{high}}}} { } - {\text{CI}}_{{{\text{low}}}} }}{2t},$$

where CI_high_ and CI_low_ are the upper and lower limits of the CIs, *n* is the group sample size, and *t* is the value of the *t* distribution with *n–*1 degrees of freedom and 95% level of confidence. To calculate the effect size, the difference of the means (post-intervention—pre-intervention for each group) and the SD of the difference between pre and post were required. To calculate the SD of the difference, the Pearson correlation coefficient (*r*) between the raw values at the two measurement times is needed, but it is rarely reported in the studies. Therefore, a standard *r* value of 0.8 was used, using the more conservative *r* value according to Mattioni Maturana et al. [34]. The SD of the difference was then calculated as:

$${\text{SD}}_{{{\text{diff}}}} = \sqrt {{\text{SD}}_{{{\text{pre}}}}^{2} + {\text{SD}}_{{{\text{post}}}}^{2} - 2r \times {\text{SD}}_{{{\text{pre}}}} \times {\text{SD}}_{{{\text{post}}}} } ,$$

where SD_diff_ is the SD of the difference and SD_pre_ and SD_post_ are the SDs at the two measurement times. Within-group Cohen’s effect size (*d*) was calculated as:

$$d = \frac{{M_{{{\text{post}}}} - M_{{{\text{pre}}}} }}{{SD_{{{\text{diff}}}} }}.$$

When a control group was present, between groups (i.e. HIT vs CON [moderate-intensity continuous training {MICT} or non-exercise control]), Cohen’s *d* was either retrieved from the original studies or—if missing or different from Cohen’s *d*—computed, considering the homoscedasticity assumption, as:

$$d = \frac{{M_{{\text{diff HIT}}} - M_{{\text{diff CON}}} }}{{\sqrt {\left[ {\frac{{\left( {n_{{\text{ HIT}}} - 1} \right)\left( {{\text{SD}}_{{\text{diff HIT}}}^{2} } \right) + \left( {n_{{\text{ CON}}} { } - 1} \right)\left( {{\text{SD}}_{{\text{diff CON}}}^{2} } \right)}}{{n_{{\text{ HIT}}} + n_{{\text{ CON}}} - 2}}} \right]} }},$$

where *M*_diff_ and SD_diff_ are the mean and SD of the changes over time (i.e. post—pre values) of the outcomes of interest and *n* is the sample size for each group. The interpretation of effect size was based on the benchmarks suggested by Cohen [35] as trivial (*d* < 0.2), small (*d* = 0.2–0.5), medium (*d* = 0.5–0.8), or large (*d* > 0.8). For one study [36], the effect size for the proportion was computed [35].

Finally, feasibility data extraction was conducted by considering participants’ attendance rates (i.e. adherence and dropouts), their perceptions (i.e. participants’ opinions on positive and negative aspects of the intervention), and the intervention fidelity (i.e. measures of whether the intervention was delivered as intended).

### Methodological Quality Assessment

The Physiotherapy Evidence Database (PEDro) scale was used to assess the risk of bias and the methodological quality of the included studies [37]. The PEDro scale rates studies with a 0–10 scale; studies with scores ≥ 6 are considered high-quality, those with scores of 4–5 are considered moderate quality, and those with scores ≤ 3 are considered low quality. Additionally, two particular criteria were utilised to judge the quality of the studies in the presence of process evaluation, determining whether (1) intervention fidelity (the intervention was given to all participants consistently and as intended) and (2) adherence to the protocol were evaluated. One author conducted study evaluations, and a second author double-checked the scores assigned. The evidence of the effectiveness of each study was used in combination with the quality score for the discussion of the results.

## Results

### Study Characteristics

**Fig. 2:**
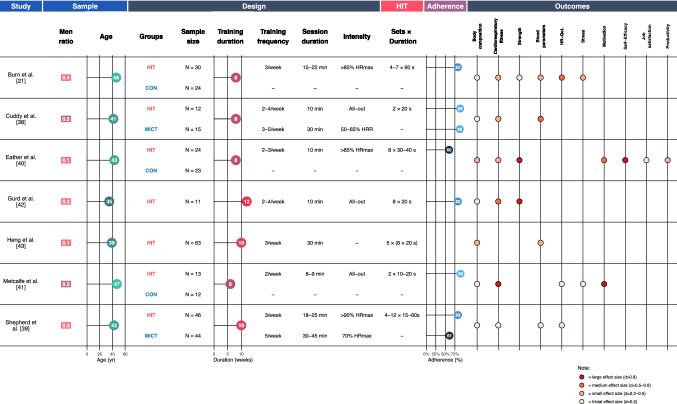
GOfER diagram of the studies' characteristics and main results. Mean age and adherence rates are reported. If two groups were present, age was reported as the median between the two groups. For graphical reasons, outcomes are summarised into categories: each category might contain more than one outcome (e.g. “body composition” includes outcomes such as weight, body mass index, fat mass). *Circles* represent the between-group effect sizes (if a control group was present) or within-group effect sizes (in the case of a pre–post design without a control group), representing the highest effect size value recorded in each category. *CON* control group, *d* Cohen’s *d* effect size, GOfER Graphical Overview for Evidence Reviews, *HIT* high-intensity interval training, *HRmax* maximal heart rate, *HR-QoL* health-related quality of life, *HRR* heart rate reserve, *MICT* moderate-intensity continuous training, *–* not reported

The literature searches identified a total of 496 articles. After the screening and selection process, seven studies with a total of 317 participants met the inclusion criteria (Fig. 1). Of these, two studies were RCTs [38, 39], one was a pilot RCT [40], and one was a mixed-methods pilot trial [21], while the remaining three were randomised [41] and non-randomised [42, 43] feasibility studies. One study [39] reported the feasibility results in a separate subsequent paper [44]. The studies included male and female adult workers (range of mean age 35–47 years), mainly employed in universities, hospitals, or office settings. Two studies [38, 39] reported that participants were physically inactive prior to the intervention, one study [41] categorised them as low-to-moderately active (based on International Physical Activity Questionnaire [IPAQ] scores), while Eather et al. [40] reported that participants identified themselves as “sedentary at work”. The three remaining studies did not specify the physical activity level of the participants; however, workers enrolled in Burn et al. [21] could be considered as sedentary based on their baseline maximal oxygen consumption (*V*O_2max_) values (≈ 37 ml/kg/min), and Heng et al. [43] enrolled overweight/obese individuals. Interventions lasted between 6 and 12 weeks, with a frequency of 2–4 HIT sessions/week for a session duration of 8–30 min (median = 10 min). HIT protocols varied between the studies: three studies [38, 39, 41] included sprints with durations ranging from 10 to 60 s and work:rest ratios from 1:2 (e.g. 60 s work, 120 s rest) to 1:9 (e.g. 20 s work, 180 s rest); two studies [42, 43] applied a Tabata protocol, which consists of eight 20-s efforts interspersed by 10 s rest; other HIT protocols included intervals ranging from 30 to 60 s, with work:rest ratios of 1:0.5 (i.e. 40 s work, 20 s rest), 1:1 (i.e. 30 s work, 30 s rest), or 1:1.25 (i.e. 60 s work, 75 s rest), repeated 4–8 times [21, 40]. Due to the heterogeneity in interventions’ characteristics and outcome measures, a meta-analysis was not feasible; thus, a narrative synthesis was conducted. A summary of the selected studies is reported with a Graphical Overview for Evidence Reviews (GOfER) diagram in Fig. 2 [45].

### Feasibility

Feasibility was assessed by considering participants’ attendance, their perceptions, and the intervention fidelity, when available.

#### Attendance

Adherence rates mostly ranged between 83 and 90%, except for Eather et al. [40], which reported 60% attendance despite the lowest dropout rate (4%), and one study [43] not reporting information on it (Fig. 2). The two studies which had an exercising control group [38, 39] reported comparable attendance values between the MICT and the HIT groups, particularly when considering attendance at the supervised exercise sessions provided in the two training modalities [39]. Not only exercise supervision, but also the weekly frequency and the duration of the training, should be considered when considering adherence and dropout rates. The latter showed high variability across the studies, ranging between 4% [40] and 65% [43]. When exploring the reasons given by participants who dropped out, the motives were usually not related to the intervention itself: personal (e.g. pregnancy, family bereavement, unrelated illness, or injuries) and work-related reasons (e.g. work accident, relocation by employers) were the most frequently reported [21, 38, 39, 41]. In the studies involving Tabata protocols as the HIT intervention [42, 43], participants’ inability to meet the time commitment and unsatisfactory participation were the main reasons for abandoning, but no further details were provided. Gurd et al. [42] hypothesised that the absence of social aspects during the intervention might have been a further reason for the low adherence rates. Notably, the MICT control group involved in the Shepherd et al. [39] study showed a considerably higher dropout rate (22%) than the HIT group (9%), which is in line with the participants’ time commitment (30–45 min, 5 times/week vs 18–25 min, 3 times/week).

#### Participant Perceptions

Four of the seven studies collected participants’ perceptions of HIT interventions through qualitative methods by using ad hoc questionnaires [40], individual interviews [41], or focus groups [21, 44]. The most frequently reported positive aspects across all four studies were considered in the time “domain” of the protocols; in particular, participants were satisfied with the time efficiency offered by the combination of several aspects such as a flexible time schedule, weekly frequency, the short session duration, and the location (proximity to the workplace). Generally, a favourable perception of exercise bouts was reported, with positive feelings of being energised, a sense of competence and achievement related to “individual nature” intensity, enjoyment, and socialisation. These positive affects were reported as possibly counteracting the negative feelings perceived during the protocol's latter stages (i.e. discomfort, anxiety, and uneasiness) as the exercise duration increased [41]. Another relevant characteristic concerned exercise monitoring in the form of instant feedback given by both an exercise specialist supervisor [21, 44] or an automated system of monitoring [41]. Finally, major beneficial outcomes were recognised across all studies in the physical and mental domains of health, as well as an increased intention to maintain physical activity habits beyond the intervention [21, 41]. When asked about barriers or less satisfactory aspects of the protocols, participants agreed on specific exercise types (i.e. stair climbing, stepping, or boxing) [21], or the strenuous nature of effort that in a few cases contributed to participants’ reluctance to continue HIT exercise in the workplace [41] or in a public gym context [44].

#### Intervention Fidelity

The fidelity of the intervention—whether the intervention was delivered as intended in a comparable manner to all participants—was explicitly evaluated in three studies [21, 40, 41]. For this purpose, the intervention implementation’s acceptance was evaluated using heart rate responses [21, 40] and mean peak power output [41] during exercise to monitor the intensity. When participants’ maximum heart rate (HR_max_) was used, the fidelity of the intervention was maintained, showing averaged values of 86% [40] to 87% [21] of HR_max_ across all the exercise sessions, when the target heart rate for high-intensity exercise was set at > 85% HR_max_. During all-out sprints, participants achieved peak power outputs approximately 2.8-fold higher than those they achieved during the *V*O_2max_ test [41], maintaining fidelity to the protocol although it was delivered unsupervised.

### Effectiveness

#### Physical Fitness Outcomes

Among the health-related physical fitness components [46], studies focused on body composition, cardiorespiratory, and muscular strength measures. The effects of HIT interventions on body composition measures (e.g. body mass, fat mass) were investigated by all seven studies included, and trivial-to-small effect sizes were reported. Eather et al. [40] reported a small effect size for body mass index (BMI) when comparing the HIT group to a non-exercising control group (*d* = 0.40), with both groups showing an increase. Heng et al. [43] reported a small effect size for a reduction (*d* = 0.31) of the BMI in the HIT group, but did not include a control group. Only the studies by Cuddy et al. [38] and Shepherd et al. [39] compared the HIT intervention with an MICT group, with both studies reporting trivial effect sizes between the groups in fat mass changes (*d* = 0.03 for both).

Six out of seven studies investigated the CRF of the participants, reporting trivial to large effect sizes. The study conducted by Metcalfe et al. [41] reported a large between-group effect size (*d* = 1.4) for *V*O_2max_ improvement when comparing the HIT and non-exercising control groups. A medium within-group effect size was reported by Gurd et al. [42] (*d* = 0.74) in the improvement in aerobic fitness measured with the modified Canadian Aerobic Fitness Test. Burn et al. [21] and Cuddy et al. [38] reported small between-group effects for *V*O_2max_ improvement when comparing the HIT group with a non-exercising control group (*d* = 0.47) and an MICT group (*d* = 0.37), respectively. A small between-group effect size was also reported by Eather et al. [40] for improvement in CRF assessed by a 20-m shuttle run test (*d* = 0.34). Shepherd et al. [39] reported a significant increase in *V*O_2max_, even without differences between the HIT and MICT groups (*d* = 0.09).

Three studies included muscular fitness measures. Eather et al. [40] reported large between-group effects both for upper-limb (*d* = 0.95) and lower-limb (*d* = 1.12) muscular strength when comparing the HIT group with the non-exercising control group. Gurd et al. [42] reported large within-group effect sizes for the upper limbs (*d* = 0.96) and medium effect sizes for the lower limbs (*d* = 0.80) regarding muscular strength. Conversely, Burn et al. [21] reported trivial effects on upper- and lower-limb muscular strength between HIT and control groups (*d* ranging from 0.08 to 0.20).

#### Cardiometabolic Health Outcomes

Cardiometabolic health was taken into consideration by four out of seven studies, which reported small-to-medium effect sizes. The variables collected were blood pressure and haematological parameters. The effects of HIT in the workplace on blood pressure were heterogeneous. Cuddy et al. [38] reported a medium effect size (*d* = 0.53) between the HIT and MICT groups, with the former showing a greater decrease in systolic pressure, while no difference was detected for diastolic pressure. Conversely, Shepherd et al. [39] reported a medium effect size for systolic pressure (between-group *d* = 0.52), with a reduction in the MICT group only; however, it should be noted that at baseline this group had higher systolic blood pressure values compared to the HIT group. Burn et al. [21] reported a trivial effect size of HIT intervention compared to the non-exercising control group, both for systolic (*d* = 0.09) and diastolic blood pressure (*d* = 0.01).

Regarding the lipid profile, Cuddy et al. [38] reported small effect sizes for an increase in high-density lipoprotein (HDL) cholesterol (*d* = 0.28) and a decrease in triglycerides (*d* = 0.31) in the HIT group compared to the MICT group. Similarly, Shepherd et al. [39] found a significant increase in HDL cholesterol, with a small effect size in favour of the MICT group (*d* = 0.39), and a reduction in low-density lipoprotein (LDL) cholesterol, without differences between groups. Likewise, Heng et al. [43] reported a small reduction effect in LDL and total cholesterol following the HIT intervention (*d* = 0.29 for both variables). Conversely, Burn et al. [21] reported a small effect size for a reduction of HDL cholesterol (*d* = 0.20) in the HIT group compared to the non-exercising control group, while a trivial effect on triglycerides was reported (*d* = 0.09).

#### Psychological and Work-Related Outcomes

The psychological and work-related outcomes were less thoroughly investigated in the studies; three studies measured the HR-QoL and two studies the stress levels. Burn et al. [21] reported medium effect sizes of the intervention among HR-QoL domains between the HIT and non-exercising control groups, with increased vitality (*d* = 0.51) and reduced pain (*d* = 0.67); small effect sizes were also reported for improved perceived general health (*d* = 0.35) and stress (*d* = 0.40). Likewise, significant improvements in perceived health and subjective vitality in both HIT and MICT groups were also reported by Shepherd et al. [39], although with trivial effect sizes between groups (*d* = 0.14 and *d* = 0.03, respectively). An improvement in the general health domain of the HR-QoL after the intervention was also reported by Metcalfe et al. [41], with a trivial effect between the HIT group and control group (*d* = 0.11); no differences in perceived stress were reported. Moreover, this study reported a large between-group effect size for increased autonomous motivation to exercise (*d* = 0.88). In addition, a medium between-group effect for improved autonomous motivation to exercise (*d* = 0.76) and a large between-group effect for self-efficacy (*d* = 1.57) were reported by Eather et al. [40] between the HIT and the non-exercising control groups. In addition, this was the only study investigating work-related outcomes: a small effect for work productivity (*d* = 0.47), which improved in the HIT group, and a trivial effect for job satisfaction (*d* = 0.05) were reported.

### Quality Assessment

The methodological quality score for the seven studies, based on the PEDro scale, was a median of 5 (1st Quartile = 3; 3rd Quartile = 5.5). Due to the low number of studies selected after screening, we decided not to set a cut-off value for their inclusion in the review, but two of the seven studies scored below the suggested cut-off value of 4 (low quality) [37]. Only two studies were of high methodological quality (score ≥ 6). It should be considered that four out of the ten items of the PEDro scales are about blinding procedures, which is almost impossible to achieve in these kinds of studies. Considering the additional two criteria used to evaluate the presence of intervention process evaluation, six studies registered intervention adherence and three studies reported data for fidelity (detailed data in Sect. 3.2). Only two studies had a dropout < 15%, with one study reporting a dropout > 40%, leading to cautious interpretation of the results. The pilot trial by Burn et al. [21] did not conduct any null hypothesis testing, but effect sizes were reported. The results of the quality assessment are reported in Table 1.

**Table 1:** Qualitative assessment of the included studies

Study	Eligibility criteria	Randomised allocation	Blinded allocation	Group homogeneity	Blinded subjects	Blinded therapists	Blinded assessor	Drop out < 15%	Intention-to-treat analysis	Between-group comparison	Point estimates and variability	PEDro score	Adherence to protocol	Intervention fidelity
Burn et al. [21]	●	○	○	●	○	○	○	○	●	●	●	4	●	●
Cuddy et al. [38]	●	●	○	●	○	○	○	○	●	●	●	5	●	○
Eather et al. [40]	●	●	○	●	○	○	○	●	●	●	●	6	●	●
Gurd et al. [42]	●	○	○	○	○	○	○	○	●	○	●	2	●	○
Heng et al. [43]	●	○	○	○	○	○	○	○	●	○	●	2	○	○
Metcalfe et al. [41]	●	●	○	●	○	○	○	○	●	●	●	5	●	●
Shepherd et al. [39]	●	●	○	●	○	○	○	●	●	●	●	6	●	○

The item “eligibility criteria” is not included in the final score

*PEDro* Physiotherapy Evidence Database

● Criterium is satisfied, ○ criterium is not satisfied

## Discussion

This systematic review examined the feasibility and effectiveness of HIT interventions in the workplace setting. The main findings were that HIT can be implemented within a workplace context, and that it could be effective for improving cardiorespiratory and muscular fitness and in producing positive changes in psychological outcomes.

### Feasibility

The first concern regarding HIT implementation in the workplace was its feasibility; indeed, promoting practical, feasible, and enjoyable exercises is fundamental to maximising participants' adherence and compliance with the training protocol and thus achieving the desired results [21]. This point has been debated in the context of public health [28], the argument being that, despite its recognised efficacy in improving CRF under optimally controlled circumstances, HIT will not work in the real setting because of its negative impact on the affective response, especially for non-trained people. Three of the selected studies investigated this aspect, and one study reported the feasibility results in a separate subsequent paper [39]. Moreover, it should be kept in mind that the results pertain to small sample size studies (generally subgroups of all participants) for a quite short period of time, thus not leading to a clear indication of feasibility and participants’ opinions. The most relevant topics identified for HIT as a feasible strategy for workplace exercise were its time-appealing nature, the positive feelings derived from participation in a similar-abilities group, the supervision of the instructor and the possibility of monitoring the progress of the training. These perceived advantages contributed to increasing participants’ intention to participate in and maintain exercise. Notably, in the qualitative investigation by Kinnafick et al. [44], participants experienced increased self-competence as well as an increased self-efficacy to perform HIT, as was also found by Eather et al. [40]. Despite targeting only healthy adult employees, this constitutes a promising result in workplace feasibility; indeed, self-efficacy, defined as beliefs about one's own capabilities to plan and execute a specific behaviour [47], was consistently associated with adopting and maintaining physical activity behaviour in healthy adults [48] and, more specifically, in workplace health promotion [49, 50]. Moreover, when an intervention technique is associated with a change in effect sizes for self-efficacy, it also tends to be associated with a change in effect size for physical activity levels [51, 52], implicating adherence to the intervention. Relatedly, improved motivation to exercise (e.g. autonomous motivation, desire to exercise, and autonomy for exercising) was found with workplace HIT [40, 41] interventions among healthy employees of different contexts (university and local government offices), offering a promising finding for exercise maintenance [53]. In the opposite direction from these results, interventions presented by Gurd et al. [42] and Heng et al. [43] reported the highest dropout rates, without reporting data on fidelity or participants’ opinions. The Tabata protocol implemented in these studies possibly elicited negative affective responses, as shown in Follador et al. [54]; moreover, in Heng et al. [43], overweight employees were involved, and the high-intensity exercise could have exacerbated a negative affective response during the intervention.

### Effects on Weight and Body Composition

The studies’ results regarding body composition, which show trivial-to-small effect sizes, appear questionable, as only one [43] out of seven studies that explored these variables reported beneficial changes in body composition. However, when considering the participants’ characteristics, they were overweight or obese, meaning improvements in weight were desirable and likely easier to obtain [55]. The results obtained in the present review are in line with available literature obtained in other settings, which shows that HIT was able to decrease total body fat solely in individuals with an excess of adiposity [56, 57]. Indeed, when a relatively heterogeneous sample of studies (i.e. including active and sedentary individuals and apparently healthy and clinical populations) is considered, a trivial overall effect size in the change of body composition between HIT and MICT was reported [34, 58], with several studies reporting no significant changes in body composition after the HIT interventions [34]. Therefore, HIT should be further investigated as an effective strategy for weight management, especially in overweight or obese individuals, possibly lowering the risk of weight gain and counteracting the high BMI generally reported in workers with a prolonged sitting time [59]. Moreover, HIT was previously associated with an increased motivation to eat healthier, which might further facilitate weight control [60].

### Effects on Cardiorespiratory and Muscular Fitness

The efficacy of HIT in improving cardiorespiratory and muscular fitness in the sedentary population is not—and will not be—a primary result of this review, as many others have already reached an agreement on this [61, 62]. However, the studies included here support the accepted idea that HIT is an effective training modality for improving CRF (i.e. *V*O_2max_), also in the workplace setting. Five out of the six studies that measured, directly or indirectly, the CRF reported improvements post-intervention. The mean improvement in *V*O_2max_ was approximately 10% in the studies that directly measured it [21, 38, 39, 41]. The lowest improvement was found by Metcalfe et al. [41], who reported a mean increase of 7.4%. It should be noted that this study had a shorter intervention period (6 weeks) and a lower training frequency (2 sessions/week) than the other studies; thus, it could be speculated that greater improvements would be seen with a longer intervention. Despite the heterogeneity of between-group effect sizes, the studies reported mean changes in relative *V*O_2max_ ranging from 2.8 to 4.7 ml/kg/min; given that every increase in *V*O_2max_ of 1 ml/kg/min has been associated with a 45-day increase in longevity [63], the results of these interventions might have led to a gain of approximately 95 to 210 days of life. An improvement of 3–4 ml/kg/min in *V*O_2max_ has also been associated with a reduction in cardiovascular mortality (− 19%) [64].

For the outcomes relative to muscular fitness, the results seem more consistent: three out of seven studies included some form of muscular fitness measurements (i.e. handgrip strength, push-ups, vertical jump, squat, leg and back extensor strength), and two of them reported large effect sizes in improvements of the measured parameters [40, 42]. Muscle strength has been associated with functional ability, HR-QoL [65, 66], and job performance [67]. Furthermore, it has also been associated with a reduction in musculoskeletal disorders [14], which are among the most common causes of absenteeism and loss of productivity, resulting in high costs to employers [68]. It was highlighted that strategies developed to prevent and reduce the incidence of such problems should be included in every health-related workplace intervention [14]; however, further studies are needed to investigate the effect of HIT on musculoskeletal disorders reduction, as none of the studies included in this review considered it as an outcome.

### Effects on Cardiometabolic Health

Four of the included studies [21, 38, 39, 43] investigated the effects of HIT interventions on blood pressure and other haematochemical parameters, showing trivial-to-medium effects in lowering both systolic and diastolic pressure, and trivial-to-small effects in regulating triglycerides and cholesterol levels. The results of the present review are coherent with those reported by Mattioni Maturana et al. [34], which point out that workplace interventions based on HIT can be effective for reducing blood pressure; however, their effectiveness seems to be dependent on baseline blood pressure levels. Indeed, it is noteworthy that Burn et al. [21] and Cuddy et al. [38], whose participants’ baseline systolic blood pressure values were slightly elevated (on average approximately 129 mmHg), reported larger reductions of systolic blood pressure after HIT interventions; in another study, Shepherd et al. [39], whose HIT group participants’ baseline systolic blood pressure values were lower (on average around 123 mmHg), reported no effects of the HIT intervention.

The results obtained regarding the effects of workplace HIT interventions on the lipid profile are also in line with the studies selected by Mattioni Maturana et al. [34], which reported trivial between-group effects both for increase in HDL cholesterol and decrease in LDL cholesterol levels. These findings are in accordance with previously reported results, which suggested that low volume (< 15 min) HIT might lead to similar cardiometabolic improvements (such as glucose control, blood pressure, and cardiac function) when compared to higher volume continuous training, despite a reduced time requirement and lower energy expenditure [69]. Workers’ cardiometabolic health could have a strong impact on work productivity: indeed, a study conducted in 2007 in the USA highlighted that those individuals with higher cardiometabolic risk factors missed 179% more workdays than their healthy colleagues, resulting in a loss of productivity of 17.3 billion dollars per year [70].

### Effects on Psychological Outcomes

Four of the selected studies [21, 39–41] explored the effect of workplace HIT interventions on psychological outcomes, reporting trivial-to-medium improvements in HR-QoL and its subscales (vitality, general health, mental wellbeing), in accordance with other studies in the literature, which reported similar findings following HIT [71]. Moreover, part of this effect could be explained by the group-based nature of HIT interventions, as social interactions during exercise have been shown to positively influence overall quality of life compared to exercising alone [72]. In the same vein, the medium-to-large effects found for increased autonomous motivation to [40, 41] and self-efficacy in exercise [40] corroborate the preliminary evidence reported for HIT in improving motivation and intention to maintain exercise behaviours in different populations [73, 74].

Two studies [21, 41] investigated the effects of HIT on stress reduction, but only one found improvements in this parameter [21]. High stress levels, together with anxiety, and depression represent important public health issues, and have been associated with reduced productivity [75], increased absenteeism [76], and lower job performance [77]. Further, physically inactive workers are more likely to show higher stress levels and symptoms of burnout [78]. The evidence for the effect of HIT on work-related outcomes is limited; only one study included such measures [40], reporting trivial and small effects for improvement in job satisfaction and productivity. The relationship between physical activity and work-related outcomes has often been inconclusive, although some evidence suggest positive effects of physical activity on work performance, reduced absenteeism, and sick leave [79]. Absenteeism has been inversely associated with the number of exercise days [80] and participation in sports activities [81], while Pronk et al. [82] reported a positive association between CRF and work performance. Moreover, an inverse dose–response relationship was reported between vigorous-intensity physical activity and the number of sick leave days, but this relationship was not present with moderate-intensity activity [83]. These results would support the idea of preferring low-volume high-intensity over MICT for the implementation of physical activity in the workplace.

### Recommendations for Future Research and Limitations

The analysed studies had several limitations. Two of the seven studies were pilot trials, and the sample size was relatively small in most of the studies. Heterogeneity was present among the baseline physical activity levels of the participants, with some studies defining them as sedentary, some as physically inactive, and others not reporting this information. Participants’ characteristics might strongly influence their responses to exercise. Furthermore, the studies included only white-collar workers (university, hospital, office) who conduct sedentary work; to the authors’ knowledge, no studies are present at the moment on the effects of HIT on blue-collar workers, who already perform physically demanding tasks on a daily basis. Another limitation, which was already acknowledged by other authors, is that several studies were conducted within a university setting, which is a highly controlled environment, and this likely increased the protocol feasibility. In addition, the studies showed considerable heterogeneity regarding the training protocols and the selected outcome measures, so it was not possible to compare them by conducting meta-analytic statistics. Only two studies were RCTs, and another two studies had low quality, mainly due to the lack of a control group, and even where a comparison group was present, it was a non-exercising group; thus, it is not possible in this specific context to draw conclusions regarding higher feasibility or efficacy of HIT interventions in the workplace with respect to moderate-intensity training programmes. Acknowledging these limitations, future research should be conducted with well-designed RCTs to compare different training protocols and determine the best combination of variables (e.g. mode, frequency, duration, intensity) able to maximise the feasibility and efficacy of exercise-based interventions in the workplace. Furthermore, participants included in the analysed studies were healthy (except one that included overweight/obese individuals, despite them being considered physically fit for exercising); future research could focus attention on the proportion of employees who have some kind of musculoskeletal or metabolic disorder to determine whether such individuals would derive even greater benefits from HIT practice. Lastly, only one study attempted to investigate the effects of HIT on work-related performance. Health-promotion programmes might allow employers economic savings (US$3.48 and US$5.82, respectively, for health care and absenteeism, per each dollar invested in health promotion interventions) [84], and in addition, healthy workers have been shown to be 4–10% more productive than non-healthy ones [85]. Consequently, future studies should implement measures to estimate the financial impact of exercise-related interventions on companies and raise the awareness of the employers about workers’ health.

## Conclusion

Exercise interventions in the workplace represent a viable approach to increasing employees’ health. However, many of these still fail, mainly due to poor integration in the work environment, perceived lack of time, low self-efficacy, and lack of motivation. In this context, HIT might represent a feasible strategy to implement physical exercise in the workplace setting due to its time efficiency, the variety of exercises that can be proposed, its group-based nature, and the positive feelings of achievement raised in the participants. Some promising results emerged for HIT in improving both muscular strength and cardiovascular fitness, enhancing perceived quality of life and the motivation to exercise, and reducing stress levels. However, small sample sizes, short intervention durations, and a lack of rigorous study designs strongly affect the possibility of drawing firm conclusions on workplace HIT feasibility and effectiveness. Despite evidence for effectiveness being still weak, the reported increased self-efficacy and motivation to exercise among participants might play a key role in promoting long-term adherence to cost-effective HIT initiatives in the workplace and, likely, facilitating consistent physical, psychological, and work-related benefits. Further well-designed studies are needed to prove the superiority of HIT over other types of exercise in the workplace setting.

## Supplementary Information

Below is the link to the electronic supplementary material.

Supplementary file1 (PDF 107 KB)

## Declarations

### Data availability statement

The datasets generated during and/or analysed during the current study are available from the corresponding author upon reasonable request.
