# Supplementary material for: Short High-Intensity Interval Exercise for Workplace-Based Physical Activity Interventions: A Systematic Review on Feasibility and Effectiveness
Source: Sports Med. 2023 Feb 25;53(4):887–901. doi: 10.1007/s40279-023-01821-4 (PMC10036456; doi:10.1007/s40279-023-01821-4)
Supplement: Supplementary file 1 — Supplementary file1 (PDF 107 KB) [file 40279_2023_1821_MOESM1_ESM.pdf]

Online Resource 1 GOfER diagram of the studies' characteristics and main within-group results.

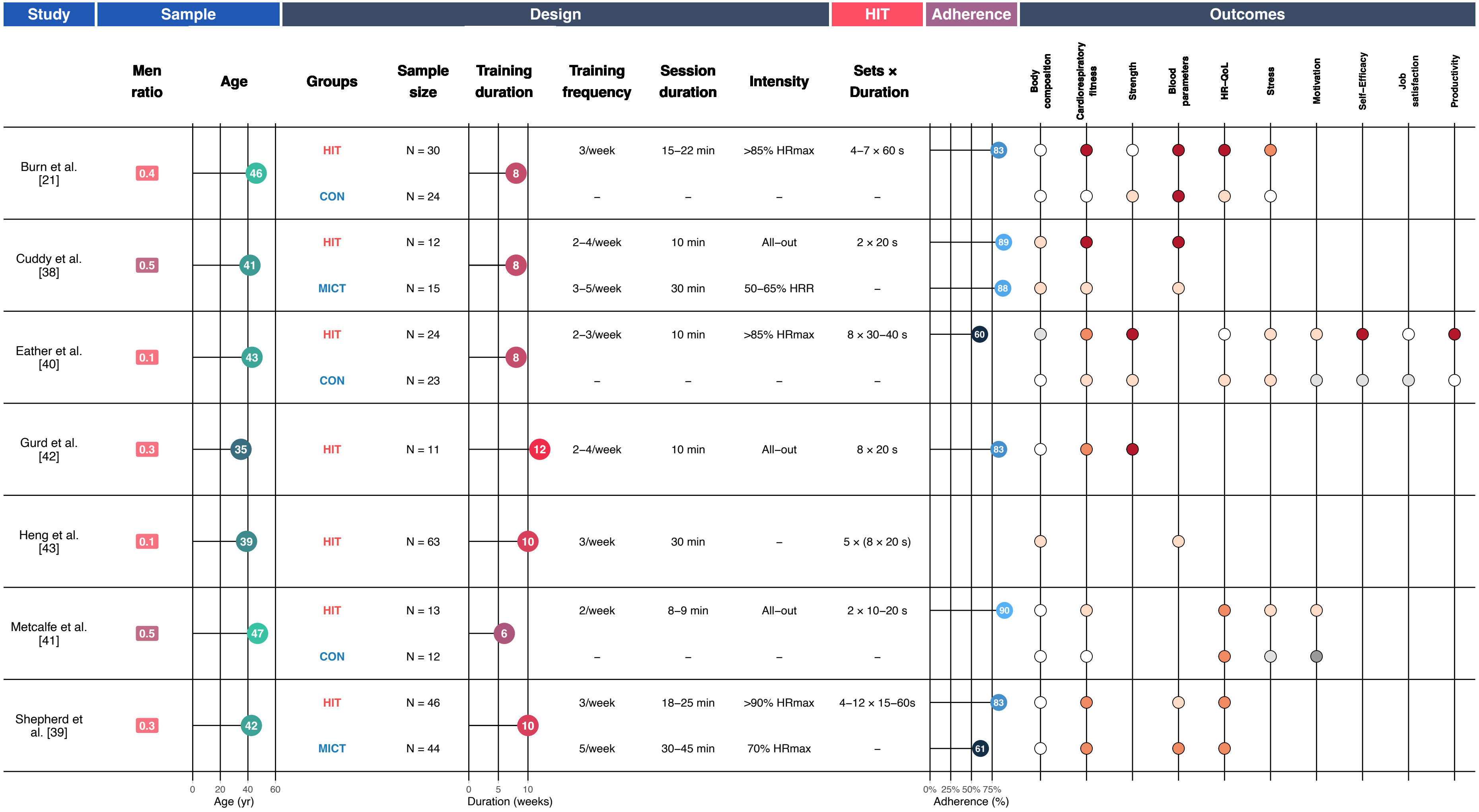

Note: Mean age and adherence rates are reported. If two groups were present, age was reported as the median between the two groups. For graphical reasons, outcomes are summarised into categories: each category might contain more than one outcome (e.g., “body composition” includes outcomes such as weight, body mass index, fat mass). Circles represent the within-group effect sizes, representing the highest effect size value recorded in each category. Circles colours indicate direction and magnitude of the effects (red = improvement, white = no effect, grey = worsening) HIT: high-intensity interval training; CON: control group; MICT: moderate-intensity continuous training; N/R: not reported; d: Cohen’s d effect size.
